# Exploration of potential biomarkers for early bladder cancer based on urine proteomics

**DOI:** 10.3389/fonc.2024.1309842

**Published:** 2024-02-12

**Authors:** Xu Zhou, Fei Xue, Tingmiao Li, Jiangshan Xue, Siqi Yue, Shujie Zhao, Hezhen Lu, Chengyan He

**Affiliations:** ^1^ Department of Laboratory Medicine, China-Japan Union Hospital of Jilin University, Changchun, China; ^2^ Department of Laboratory Medicine, Changchun Infectious Diseases Hospital, Changchun, China

**Keywords:** bladder cancer, proteomic, urine, biomarker, ELISA

## Abstract

**Background:**

Bladder cancer is a common malignant tumor of the urinary system. The progression of the condition is associated with a poor prognosis, so it is necessary to identify new biomarkers to improve the diagnostic rate of bladder cancer.

**Methods:**

In this study, 338 urine samples (144 bladder cancer, 123 healthy control, 32 cystitis, and 39 upper urinary tract cancer samples) were collected, among which 238 samples (discovery group) were analyzed by LC−MS. The urinary proteome characteristics of each group were compared with those of bladder cancer, and the differential proteins were defined by bioinformatics analysis. The pathways and functional enrichments were annotated. The selected proteins with the highest AUC score were used to construct a diagnostic panel. One hundred samples (validation group) were used to test the effect of the panel by ELISA.

**Results:**

Compared with the healthy control, cystitis and upper urinary tract cancer samples, the number of differential proteins in the bladder cancer samples was 325, 158 and 473, respectively. The differentially expressed proteins were mainly related to lipid metabolism and iron metabolism and were involved in the proliferation, metabolism and necrosis of bladder cancer cells. The AUC of the panel of APOL1 and ITIH3 was 0.96 in the discovery group. ELISA detection showed an AUC of 0.92 in the validation group.

**Conclusion:**

This study showed that urinary proteins can reflect the pathophysiological changes in bladder cancer and that important molecules can be used as biomarkers for bladder cancer screening. These findings will benefit the application of the urine proteome in clinical research.

## 1 Introduction

Bladder cancer is the sixth most common malignant tumor in America (1), and its incidence rate ranks seventh among male malignant tumors in China (2). According to existing reports, its pathogenesis is mainly associated with smoking (3), ethnicity (4), occupational exposure to heavy metals such as arsenic and cadmium (5), etc. The main clinical manifestation is painless gross hematuria. When the lesions involve the bladder trigone, the “bladder irritation sign”, namely, frequent urination, urgency, and dysuria, is evident. In terms of prognosis, the survival of bladder cancer patients mainly depends on the tumor stage (6), and the tumor stage is determined based on the presence or absence of muscle invasion. The prognosis of patients with an advanced muscle invasive type of bladder cancer is poor (7), with a 5-year survival rate of only 15% (8). Therefore, early diagnosis and improvement of the radical surgical resection rate are particularly important for patients with bladder cancer. Consequently, a simple and convenient detection method is urgently needed to diagnose bladder cancer.

Screening methods for bladder cancer include urine exfoliation cytology, cystoscopy and tumor biomarker assessment (9). Urine cytology has poor sensitivity for low-grade bladder cancer (sensitivity 25.0-48.0%) (10). Cystoscopy is the gold standard for diagnosing bladder cancer; however, it is an invasive examination that may lead to risks of bleeding, infection, etc., and easily causes patient discomfort (11). Biomarker tests for bladder cancer include CxBladder, AssureMDx, Bladder Tumor Antigen (BTA), NMP22, UroVysion and Immunocyt/uCyt+ approved by the US Food and Drug Administration (FDA). Soputro Nicolas Adrianto et al. (12) summarized the diagnostic efficacy of the above biomarker tests in the diagnosis of bladder cancer and pointed out that although some biomarkers showed good performance, they were not effective in the detection of early bladder cancer (sensitivity 65.9-97.3%, specificity 57.7-83.3%), so they could not replace cystoscopy or cytology. Therefore, in recent years, researchers have been exploring new biomarkers with both high specificity and high sensitivity for the screening of early bladder cancer.

Proteomics is one of the ways to explore biomarkers for a wide range of diseases, and the most popular studies involve cancer (e.g., hepatic carcinoma, pancreatic cancer, ovarian cancer, etc.) (13), cardiovascular disease (14), infectious diseases (e.g., COVID-19, tuberculosis, syphilis, parasitic diseases, etc.) (15), etc. For bladder cancer proteomics research, the sample source can be tissue, blood or urine. Among them, urine is stored in the bladder and is in direct contact with the urothelial cells of the bladder. Therefore, the proteins contained in urine can reflect the characteristics of bladder diseases to a great extent.

The earliest bladder cancer proteomic study, to our knowledge, was reported by Antonia Vlahou et al. (16), who conducted proteomic analysis of 94 urine samples (30 bladder cancer, 34 healthy control, and 30 benign samples) by protein chip combined with SELDI-TOF-MS in 2001. Five potential biomarkers for bladder cancer were identified (sensitivity 87.0%, specificity 66.0%). In the following 10 years, researchers carried out similar research. In 2010, Tan LB et al. (17) analyzed 55 urine samples (27 bladder cancer, 14 healthy control, and 14 benign samples) by nano-HPLC−ESI−MS/MS and identified 146 differential proteins, and PLK2 was ultimately selected as a biomarker for bladder cancer (sensitivity 80.0%, specificity 64.0%). In 2011, Rosser CJ et al. (18) analyzed 100 urine samples (54 bladder cancer and 46 controls) by LC/MS-MS and identified 265 distinct glycoproteins. 70 samples (35 bladder cancer and 35 controls) were collected for validation by ELISA and A1AT was selected as a biomarker for bladder cancer (sensitivity 74.0%, specificity 80.0%). In 2015, Kumar P et al. (19) analyzed 12 urine samples (8 bladder cancer and 4 healthy control samples) by LC−MS and conducted Western blot and ELISA verification (sensitivity 79.2-86.4%, specificity 96.7-100%) of the selected panel from 239 urine samples (110 Ta/T1, 63 T2/T3 and 66 healthy control samples). In the latest study in 2023, Tabaei S et al. (20) collected urine protein information from 42 urine samples (25 nonmuscle invasive bladder cancer and 17 muscle invasive bladder cancer samples) by 2-DE and LC-MS, and 12 differential expressed proteins were identified.

In past studies, most of the control cohorts for bladder cancer were healthy volunteers or benign disease patients, but upper urinary tract cancer patients were comparatively less involved. Furthermore, the patients with bladder cancer who do not have hematuria or other symptoms might be easy to escape diagnosis. In our study, urine samples from upper urinary tract cancer patients, cystitis patients, healthy controls and bladder cancer patients were collected. Liquid mass spectrometry (LC−MS) was used to analyze urinary proteomics to screen differential proteins as potential biomarkers for the diagnosis of bladder cancer. The potential proteins were validated by ELISA. The results of this study will benefit the application of the urinary proteome in clinical research.

## 2 Materials and methods

### 2.1 Ethical approval

The samples used in this study were from the Biobank of China-Japan Union Hospital of Jilin University. The consent procedure and research protocol for this study were approved by the Human and Animal Research Ethics Committee of China-Japan Union Hospital of Jilin University (No. 2023053015). The research methods met the standards set out in the Declaration of Helsinki.

### 2.2 Patient inclusion and exclusion criteria

The inclusion and exclusion criteria of this study were as follows: First, the test group consisted of patients with nonmuscle invasive bladder cancer, patients with nonmuscle invasive upper urinary tract cancer, and patients with cystitis glandularis. None of the patients had hematuria or bladder irritation signs, which were diagnosed by physical examination. The control group was healthy people selected from the physical examination center. Second, all patients in the test group were confirmed by postoperative pathological findings. Third, the blood biochemical indices, such as liver function and kidney function, of all people in both the disease group and healthy group were within the normal range. Fourth, nobody in the test group received any other treatment prior to surgery. Fifth, no other malignant tumors or metabolic-related diseases, such as diabetes or hyperlipidaemia, were found in the preoperative routine examination or medical history collection of all patients in the test group.

### 2.3 Sample collection

The samples in this study were the urine of tumor patients, benign disease patients and healthy controls from the Biological Sample Bank of China-Japan Union Hospital of Jilin University. None of the above patients had gross hematuria, microscopic hematuria or clinical signs, all of which were screened by physical examination.

All patients underwent cystoscopy surgical resection of the lesions, and the resected lesions were submitted to the pathology department for safekeeping. All pathological sections were jointly reviewed by 2 chief physicians or deputy chief physicians of the Department of Pathology, China-Japan Union Hospital of Jilin University, and each made a diagnosis. If the diagnosis was inconsistent, an experienced and qualified chief physician was invited to assist in the analysis, and a consistent conclusion was finally reached.

### 2.4 Sample preparation

The urine samples were thawed naturally at room temperature. After thawing, the samples were centrifuged at 5000 g and 4°C for 45 min with a thermostatic centrifuge to collect the supernatant. Three volumes of precooled acetone were added to the supernatant, and then the samples were placed in a -20°C refrigerator for 1 h. After the protein was fully precipitated, the samples were centrifuged at 14,000 × g for 30 minutes at 4°C to resuspend the pellet in lysis buffer (7 mol urea, 2 mol thiourea, 0.1 mol DTT (dithiothreitol) and 5 mmol Tris). Then, the sample was shaken, and the protein was mixed to fully dissolve. The protein concentration of the sample was calculated by the Bradford method, and the results for all proteome samples were pooled together.

The mixed samples were digested by means of the FASP method (21). Briefly, (1) 200 µg of a sample was mixed with 200 µl lysate, and then 4 µl 1 M DTT was added. After mixing, the samples were placed into a 95°C water bath for 5 min and then cooled to room temperature. (2) Then, 10 µl 1 M iodoacetamide (IAM) was added to each sample and kept at room temperature away from light for 45 min. (3) The sample was transferred to the washed 30 kDa Millipore filter and centrifuged at 14000 g and 20°C until the liquid was separated. Then, the Millipore filter was cleaned to remove impurities. (4) A total of 20 µl of 0.5 µg/µl trypsin was added to the Millipore filter; the sample was mixed well and placed in a beaker of 1 L ice water under high heat microwave for 1 min; these steps were repeated twice. (5) After 12 hours in a 37°C water bath, the filter tube was centrifuged at 20°C and 14000 × g for 10 min to recover the polypeptide solution.

### 2.5 LC-MS/MS

The sample mass spectrum signal was collected by the data-dependent acquisition (DIA) method, which was completed with an Orbitrap Fusion Lumos MS (Thermo Scientific, Waltham, MA, USA) and EASY-nLC 1000 HPLC system (Thermo Fisher Scientific, Waltham, MA, USA). To ensure the alignment of retention times between samples, iRT (Biognosys, Zurich, Switzerland) was added to all samples at a concentration ratio of 1:20. To evaluate the consistency in the results, a combined sample mixture was run every 22 samples. For the liquid phase method, mobile phase A was 0.1% formic acid aqueous solution, and mobile phase B was 0.1% formic acid aqueous solution acetonitrile. Each time, the liquid phase lasted for 60 minutes, and the flow rate was 0.3 µl/min. The liquid-phase gradient was as follows: 0-1 min, 6-11% solvent B; 1-9 minutes, 11-17% solvent B; 9-40 minutes, 17-29% solvent B; 40-50 minutes, 29-37% solvent B; 50-55 minutes, 37-100% solvent B; 55-60 minutes, 100% solvent B. The autosampler temperature was set to 4°C, and the chromatographic column was set to ambient temperature. The mass spectrum parameters were set as follows: the maximum injection time of the full scan and DIA scan was 50 ms, and the cycle time was 1.55 s. The number of precursor ions in each isolation window was equal. The full scan was set to a resolution of 120000, the m/z range was 350-1200, followed by a DIA scan with a resolution of 30000, the HCD collision energy was 32%, the AGC target was 1E6, and the maximum injection time was 50 ms.

### 2.6 Data analysis

Proteome Discoverer (PD) (version 2.1; Thermo Fisher Scientific, San Jose, CA, USA) software was used to search the data obtained, and the search results were imported into Spectronaut (Biognosys, Switzerland). The default software settings were as follows: fragment ions were selected in the range of 300-1800 m/z, the number of fragments per peptide segment was limited to 3-6, the mass tolerance of the parent ion was 10 ppm, the mass tolerance of fragment ions was 0.02 Da, and the FDR threshold was set to 1%. The peptide retention time was calculated from iRT data. Protein identification and quantification were carried out by matching retention time, m/z and other parameters with the spectral library. All results were filtered according to the Q cut-off value of 0.01 (corresponding to 1% FDR), and the protein was identified according to the two unique peptides after the Q filter. The peptide strength was calculated by summing the peak area of each fragment ion of MS2, and the protein strength was calculated by summing the strength of each peptide. Raw MS data files can be downloaded free of charge at iprox (https://proteomecentral.proteomexchange.org/cgi/GetDataset?ID=PXD043825).

### 2.7 Data pre-processing

Further data pre-processing including missing value imputation, log transformation and auto scaling were using MetaAnalyst 4.0 (http://www.metaboanalyst.ca). Variables missed in 50% or greater of the samples were removed from further statistical analysis. T-TEST was used to evaluate the significance of variables. Principal component analysis (PCA) and orthogonal partial least squares discriminant analysis (OPLS-DA) was carried out using SIMCA 17.0 (Umetrics, Sweden) software. The selected differential variables must have met p < 0.05 and fold change between two groups ≥1.5. ROC analysis and external validation were carried out using the “Biomarker discovery” module on the MetaAnalyst 4.0 platform, while logistic regression was used for machine learning.

### 2.8 Bioinformatics analysis

Functional annotation-based protein classification of biological processes, molecular functions, and cellular components was performed by Gene Ontology (GO) and Kyoto Encyclopedia of Genes and Genomes (KEGG) analyses. For Ingenuity Pathway Analysis (IPA), the investigators uploaded SwissProt accession numbers to IPA software (Ingenuity Systems, Mountain View, CA). These proteins were mapped to disease and functional classes and canonical pathways available in Ingenuity and ordered by p value.

### 2.9 ELISA detection

Two kits were used to assay human APOL1 (Bioswamp, HM11775) and ITIH3 (Bioswamp, HM12232) by double-antibody sandwich ELISA. First, gradient-diluted standard protein was added to the enzyme-linked plate, and the sample and HRP-labelled antibody were successively added to the well. Next, enzyme-labelled reagents were added to each well. After incubating at 37°C for 30 minutes, the liquid was discarded, and each well was washed 5 times with detergent. Subsequently, the developer was added. The terminating agent was added after incubating at 37°C in the dark for 10 minutes. Finally, the absorbance values of each well were measured at a wavelength of 450 nm. A standard curve based on the absorbance of the standard protein was drawn, and the protein content in each sample was calculated.

## 3 Results

### 3.1 Clinical information

A total of 338 samples were evaluated in this study, namely, 144 BC, 123 HC, 32 cystitis, and 39 UTUC samples. The healthy controls were matched by sex and age with bladder cancer patients. There was no significant difference in age, sex or disease across subjects (P>0.05).

The 338 samples were matched with gender and age before randomly divided into a discovery group (238) and a validation group (100) for subsequent analysis.

### 3.2 Workflow

**Figure 1:**
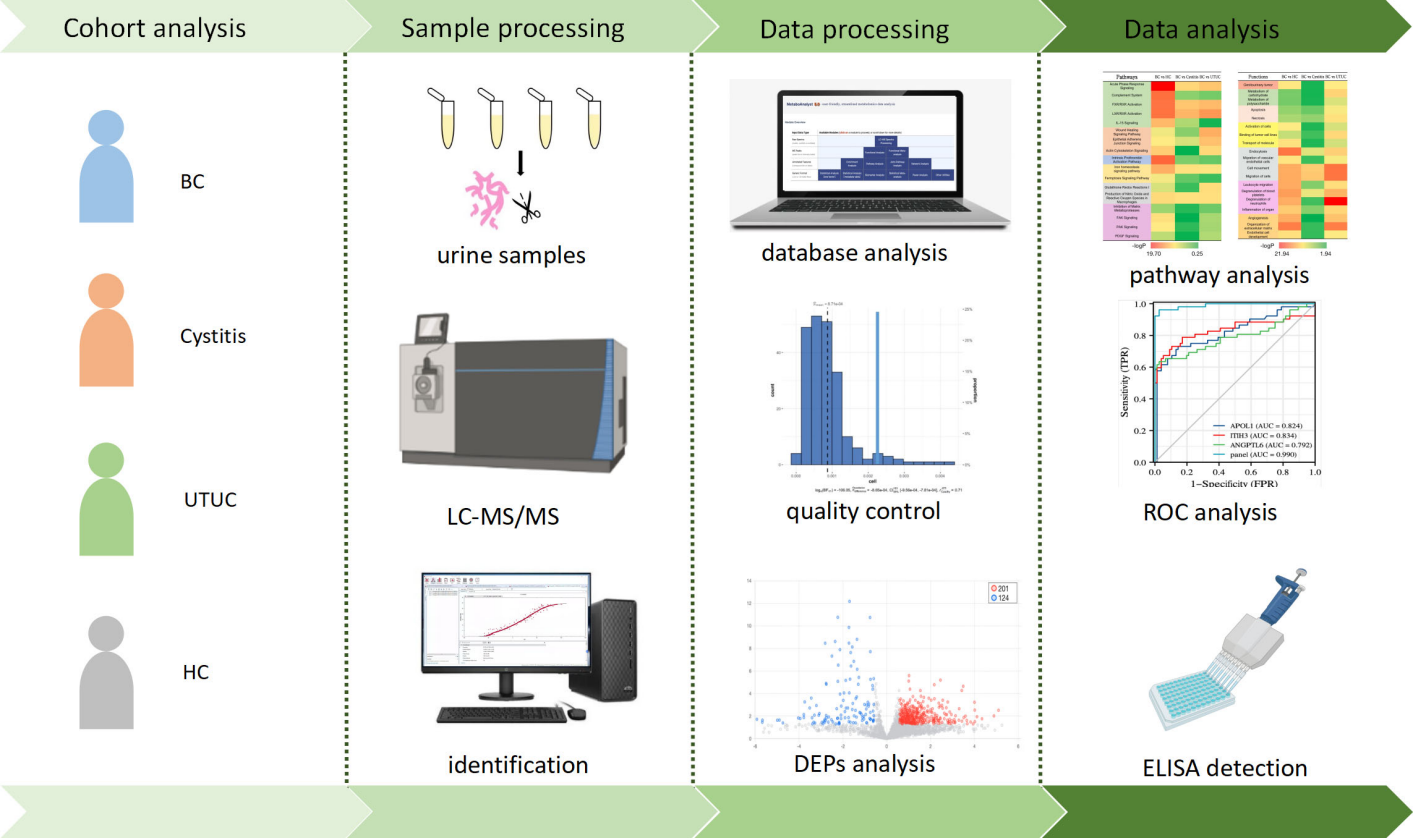
Workflow of functional analysis and biomarker screening of differential proteins in patients.

Urinary protein from each group was extracted, digested and analyzed by LC−MS. After quality control of the data, four groups were used for differential analysis, and the differential proteins were screened out. The differential proteins were used for functional annotation as well as pathway analysis. Finally, the biomarker panel was used to evaluate the prediction effect by ROC analysis and was verified in the validation group by ELISA. Finally, the potential pathological mechanism of bladder cancer was provided (**Figure 1**).

### 3.3 Establishment of bladder cancer protein library

In this study, four libraries were constructed using DIA-LC−MS technology, namely, the bladder cancer (2853 proteins), cystitis (3075 proteins), upper urinary tract cancer (3243 proteins) and healthy control (3166 proteins) libraries. Finally, a combined library of the above 4 libraries was constructed with Pulsar, including 4691 proteins. In the DIA analysis, 3331 proteins were detected with a protein FDR < 1%, of which 1871 proteins (mean=1520 per sample) of unipeptide≥2 were reserved.

### 3.4 Quality control

Initially, we conducted correlation analysis of QC samples to assess data quality and technical repeatability. The CV of protein abundance was calculated in 11 QC replicate samples. Pearson’s correlation coefficient was 0.9-1.0 (mean=0.96) in all 11 QC replicates (**Supplementary Figure S1A**), showing good technical reproducibility.

Next, we calculated the protein content of each sample in each group. Seventeen cases were excluded because their proteins were less than the mean + 2SD (**Supplementary Figure S1B**). In addition, samples suspected of blood contamination or cell debris contamination were screened by referring to a previous article (22). After calculating the proportion of blood-contaminated protein abundance and the proportion of cell-contaminated protein abundance in the samples, we excluded the samples whose abundance ratio exceeded the mean + 2SD, which were 6 and 14 cases, respectively (**Supplementary Figure S1C, D**). As a result, 201 cases remained, and this information is presented in **Table 1A**.

**Table 1A:** Patients and healthy controls (BC, bladder cancer; HC, healthy control; UTUC, upper tract urinary cancer).

Method	LC-MS				ELISA			
Group	BC	HC	Cystits	UTUC	BC	HC	Cystits	UTUC
Cases	83	75	20	23	40	40	10	10
Age	64 (28-92)	62 (28-91)	53 (22-79)	66 (48-85)	65 (31-87)	62 (30-85)	51 (34-69)	62 (51-77)
Gender (M/F)	20/63	16/59	6/14	13/10	12/28	15/25	5/5	4/6

Finally, missing values were imputed according to the k-nearest neighbor method (K-NN method) by an online data processing platform (https://www.metaboanalyst.ca). As a result, more than 50% of the samples had quantitative results (1217 proteins) for further analysis.

### 3.5 Proteomics

#### 3.5.1 Principal component analysis and orthogonal partial least squares-discriminant analysis

**Figure 2:**
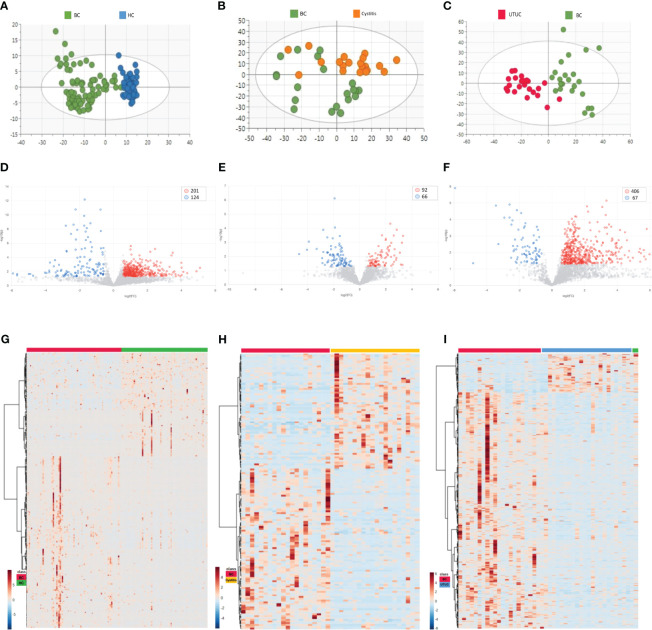
Results of PCA and differential proteins in different cohorts [**(A, D, G)** BC vs HC. **(B, E, H)** BC vs Cystitis. **(C, F, I)** BC vs UTUC.]. **(A-C)** Cluster analysis in each cohort. **(D-F)** Volcano maps in different cohorts. **(G-I)** Differential proteins in different cohorts.

First, the unbiased statistical method PCA was used to analyze the differential proteomes of bladder cancer and healthy control samples, bladder cancer and cystitis samples, and bladder cancer and upper urinary tract cancer samples. **Figures 2A-C** shows that the bladder cancer samples had a clear trend of separation from the other cohorts, with the most obvious separation from the healthy control samples. Next, OPLS-DA was performed on the three cohorts. As seen from the score chart (**Supplementary Figures S2A-C**), the bladder cancer samples were significantly separated from the healthy control, cystitis and upper urinary tract cancer samples, and the samples were highly clustered in each group. To test whether the model was overfitted, a permutation test with 100 random groups was performed. The results (**Supplementary Figure S2D-F**) showed that the clustering models of each group were not overfitted.

#### 3.5.2 Differential proteins

The criteria for fold change≥1.5 and p value<0.05 (computed with the T-Test) were used to determine differentially expressed proteins (DEPs) in the bladder cancer samples versus the other cohort samples. As shown in the volcano plots (**Figures 2D-F**) and heatmaps (**Figures 2G-I**), the numbers of DEPs were 325 (201 upregulated, 124 downregulated), 158 (92 upregulated, 66 downregulated) and 473 (406 upregulated, 67 downregulated) in the healthy control cohort, cystitis cohort and upper urinary tract cancer cohort, respectively, compared with the bladder cancer cohort (**Supplementary Table 1**).

#### 3.5.3 Results of pathway and functional annotation of differential proteins

**Figure 3:**
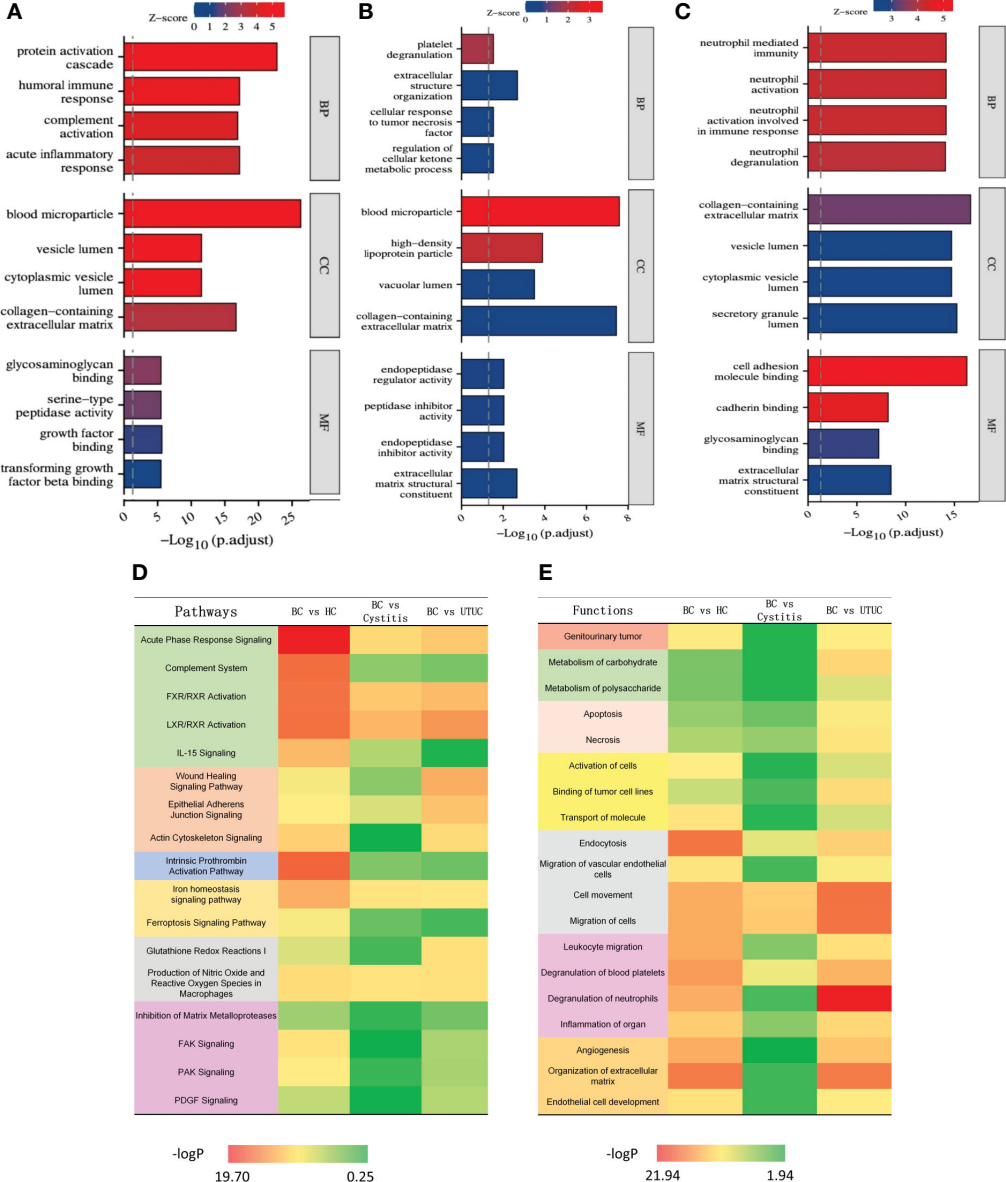
Results of KEGG and IPA analysis in different cohorts [**(A)** BC vs HC. **(B)** BC vs Cystitis. **(C)** BC vs UTUC.]. **(A-C)** Result of KEGG in each cohort. **(D)** Heat map of pathways in different cohorts. **(E)** Heat map of functions in different cohorts.

KEGG analysis was performed for the differential proteins using Xiantao Academic Online processing software (https://www.xiantao.love). The results (**Figures 3A-C**) show that the different proteins in each group were enriched in similar cell group classifications, mainly in the extracellular matrix containing collagen. However, the degree of biological process was very different. Compared with those of the healthy control, cystitis and upper urinary tract cancer samples, the proteins of the bladder cancer samples were more active in the protein-activated cascade, tumor necrosis factor and neutrophils. In molecular function, the proteins of the bladder cancer samples were more active in growth factor, regulating peptidase activity and cell adhesion function compared with the other three groups.

The pathway and functional enrichment analyses of the differential proteins among the groups were annotated by IPA, and the common parts are shown in **Figures 3D, E**. Through IPA, all of the differential proteins between bladder cancer and each group (healthy control, cystitis and upper urinary tract cancer) showed enrichment of cancer, apoptosis, proliferation, growth factors, and cytokine signaling pathways. Overall, the difference between the bladder cancer and healthy control samples was the most significant, reflecting the rapid proliferation of tumor cells relative to normal cells. The difference between the bladder cancer and cystitis samples was the least obvious, while the difference between the bladder cancer and upper urinary tract cancer samples was more significant than that between the bladder cancer and cystitis samples.

In terms of functional enrichment, similar to the results of pathway enrichment, the difference between the bladder cancer and healthy control samples was the most significant, followed by the difference between the upper urinary tract cancer and cystitis samples. Specifically, the differentially expressed proteins were significantly enriched in endocytosis and extracellular matrix function between the bladder cancer and healthy control groups. Only cell motility was significantly enriched in the differentially expressed proteins between the bladder cancer and cystitis groups. The differentially expressed proteins between the upper urinary tract cancer and bladder cancer groups were mainly enriched in neutrophils, endocytosis and cell motility. Overall, the differential proteins between the bladder cancer group and each of the other three groups (healthy control, cystitis and upper urinary tract cancer) showed enrichment of tumor, necrosis and apoptosis, migration and movement, and signal transduction. Details are shown in **Supplementary Table 2**.

#### 3.5.4 Potential biomarkers in bladder cancer and other urinary diseases

**Figure 4:**
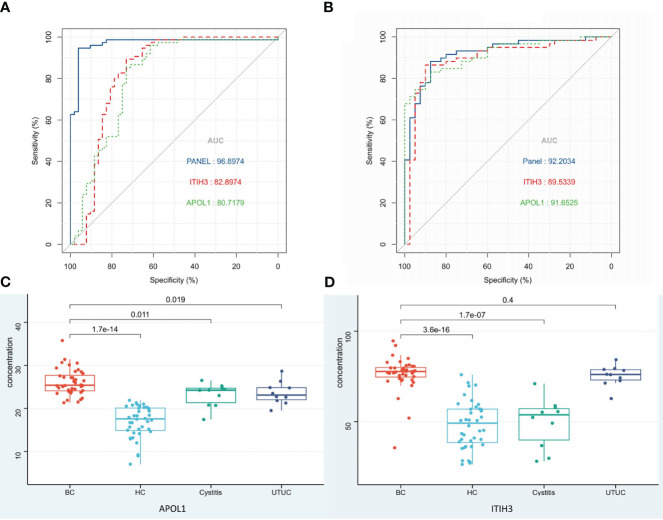
Biomarkers of BC and ROC analysis. **(A)** ROC analysis of panel and the proteins in panel (Discovery group). **(B)** ROC analysis of panel and the proteins in panel (Validation group detected by ELISA). **(C, D)** Protein expression in each group detected by ELISA.

To find specific markers for early bladder cancer, we first found the common differential proteins obtained by comparison with the cystitis, upper urinary tract cancer and healthy control groups in the above experiments and obtained 8 proteins as candidate biomarkers (**Supplementary Figure S3A**, **Table 2**). Then, after matched with gender and age, all samples were randomly divided into an discovery group and a validation group (discovery group: validation group=2:1, **Table 1B**). Next, the top 2 AUC proteins, APOL1 and ITIH3, were selected to establish a panel for ROC analysis in the discovery group(**Figure 4A**)(P<0.001), and the AUC of the panel was 0.96, which was higher than that of the individual proteins in the panel (**Table 2**). In addition, it was performed and showed the good prediction value in the validation group, with an AUC of 0.99 for the panel (**Supplementary Figure S3B**) (P=0.003). Moreover, the contents of APOL1 and ITIH3 in each group were statistically significant (**Supplementary Figures S3C, D**). Among them, the levels of APOL1 in the bladder cancer, cystitis and upper urinary tract cancer samples were higher than those in the healthy control samples. ITIH3 was higher in the bladder cancer and upper urinary tract cancer samples than in the cystitis and healthy control samples.

**Table 1B:** Patients with bladder cancer and others (HC+cystitis+UTUC) in the discovery group and validation group.

Items	Discovery Group		Validation Group	
	BC	Others	BC	Others
Cases	52	76	31	42
Age	66 (28-92)	63 (22-91)	63 (30-88)	64 (31-87)
Gender(M/F)	10/42	15/61	7/24	13/29

**Table 2:** Differentially expressed proteins between BC and others (HC + cystitis + UTUC).

Items	Name	AUC	Sensitivity	Specificity
DEFs	ITIH3	0.833(0.804-0.861)	0.825(0.796-0.853)	0.759(0.720-0.797)
	APOL1	0.820(0.793-0.847)	0.848(0.821-0.875)	0.712(0.670-0.753)
	ANGPTL6	0.774(0.744-0.804)	0.874(0.849-0.899)	0.654(0.611-0.697)
	PON1	0.670(0.637-0.703)	0.819(0.790-0.848)	0.489(0.444-0.535)
	PDL1M1	0.640(0.607-0.673)	0.556(0.518-0.593)	0.628(0.584-0.672)
	ST6GAL2	0.629(0.595-0.662)	0.501(0.464-0.539)	0.650(0.606-0.693)
	FCN3	0.577(0.541-0.614)	0.256(0.233-0.289)	0.549(0.504-0.594)
	FKBP2	0.513(0.480-0.546)	0.585(0.548-0.622)	0.393(0.394-0.437)
BC *vs* Others	Discovery Group	0.965(0.954-0.976)	0.934(0.916-0.953)	0.906(0.880-0.932)
	Validation Group	0.990(0.984-0.997)	1.000(1.000-1.000)	0.961(0.938-983)
	Test of ELISA	0.922(0.905-0.940)	0.872(0.844-0.900)	0.875(0.841-0.909)

#### 3.5.5 ELISA detection

One hundred urine samples (validation group) were used to analyze the expression levels of APOL1 and ITIH3 by ELISA (**Table 1A**, **Supplementary Table 3**). As a result, compared with LC−MS, the expression levels of two proteins showed the same tendency in ELISA results(P<0.001), and the predictive performance of the panel was similar (AUC=0.92, **Figures 4B–D**).

The concentration of APOL1 in the BC group was significantly different from that in the other groups (P<0.05). The concentration of ITIH3 in the BC group was significantly different from that in the HC and cystitis groups (P<0.05) but was not significantly different from that in the UTUC group (P>0.05), which might be due to the small sample size.

## 4 Discussion

In this study, proteomic analysis of bladder cancer was completed by LC−MS, and 331 differential proteins were also reported to be differential proteins in other studies (16–20, 23–34). The differentially expressed proteins reflected the functions of cell motility, proliferation, metabolism, necrosis and signal transduction. Moreover, we screened out a panel containing 2 proteins as potential biomarkers for the diagnosis of bladder cancer.

### 4.1 Pathway and function of bladder cancer

Proliferation and differentiation are basic biological functions of tumor cells, and the regulation of tumor proliferation and differentiation determines tumor size. In this study, many pathways regulating the proliferation and differentiation of bladder cancer were found to be active, such as the ERK/MAPK signaling pathway. It has been reported that the RAS-RAF-MEK-ERK signal chain is an important signal transduction pathway in malignant tumors (35), and in 85% of cases of low-grade early bladder cancer, MAPK is activated by mutation of the tyrosine kinase signaling pathway or Ras pathway (36). In this study, we found that the KRAS protein was upregulated in bladder cancer, suggesting that the protein may promote the proliferation and differentiation of bladder cancer cells by transducing tyrosine kinase signaling.

Migration is an important step in the development of malignant tumor cells (37), and the regulation of cell movement determines the migration ability of malignant tumor cells. In this study, we found that the actin cytoskeletal signaling pathway that regulates cell movement is active in bladder cancer. Studies have shown that the malregulation of proteins involved in this signaling pathway can affect the activity of malignant tumor cells, which rely on the actin cytoskeletal structure for migration (38). In bladder cancer, actin is depolymerized and remodeled in the cytoplasm, resulting in the transformation of bladder cancer cells and thus enhancing their migration ability (39). However, when the Ras-Rac-PAK1 pathway, which is related to the actin cytoskeleton, is inhibited, the migration behavior of tumor cells is weakened (40). In this study, the differentially expressed proteins involved in the actin cytoskeleton signaling pathway were mostly upregulated in bladder cancer, including KRAS and MAPK1 proteins involved in the ERK/MAPK signaling pathway. This suggests that the ERK/MAPK signaling pathway may have a synergistic effect with the actin cytoskeleton signaling pathway to promote the occurrence and development of bladder cancer.

Ferroptosis is a novel form of cell death often accompanied by iron accumulation and lipid peroxidation that plays a role as a dynamic tumor suppressor in cancer development (41). In bladder cancer, ferroptosis also plays a role in inducing cancer cell death and inhibiting tumor development (42), while regulating iron metabolism or cell oxidation can either induce or inhibit iron death (43). In this study, it was found that the iron homeostasis signaling pathway, glutathione redox reactions and ferroptosis pathway were all enriched in bladder cancer. Among them, the main protein involved in the iron homeostasis signaling pathway was transferrin (TF). TF was upregulated in the bladder cancer group, which may be caused by the increased iron demand of highly proliferating cells (44). Studies have found that TF upregulation can promote ferroptosis and inhibit cancer progression (45). Another pathway, glutathione redox reactions, inhibits ferroptosis by improving cellular antioxidant function (46), such as the downregulation of P53 on SLC7A11 (43). In addition, many ferroptosis inducers have been designed based on the principle of antioxidant inhibitors (47). In addition to their potential therapeutic effects, ferroptosis inducers can enhance the anticancer response of other anticancer drugs in bladder cancer (48). In general, the body may promote the death of bladder cancer cells through the ferroptosis mechanism to inhibit tumor progression and thus achieve self-protection.

**Figure 5:**
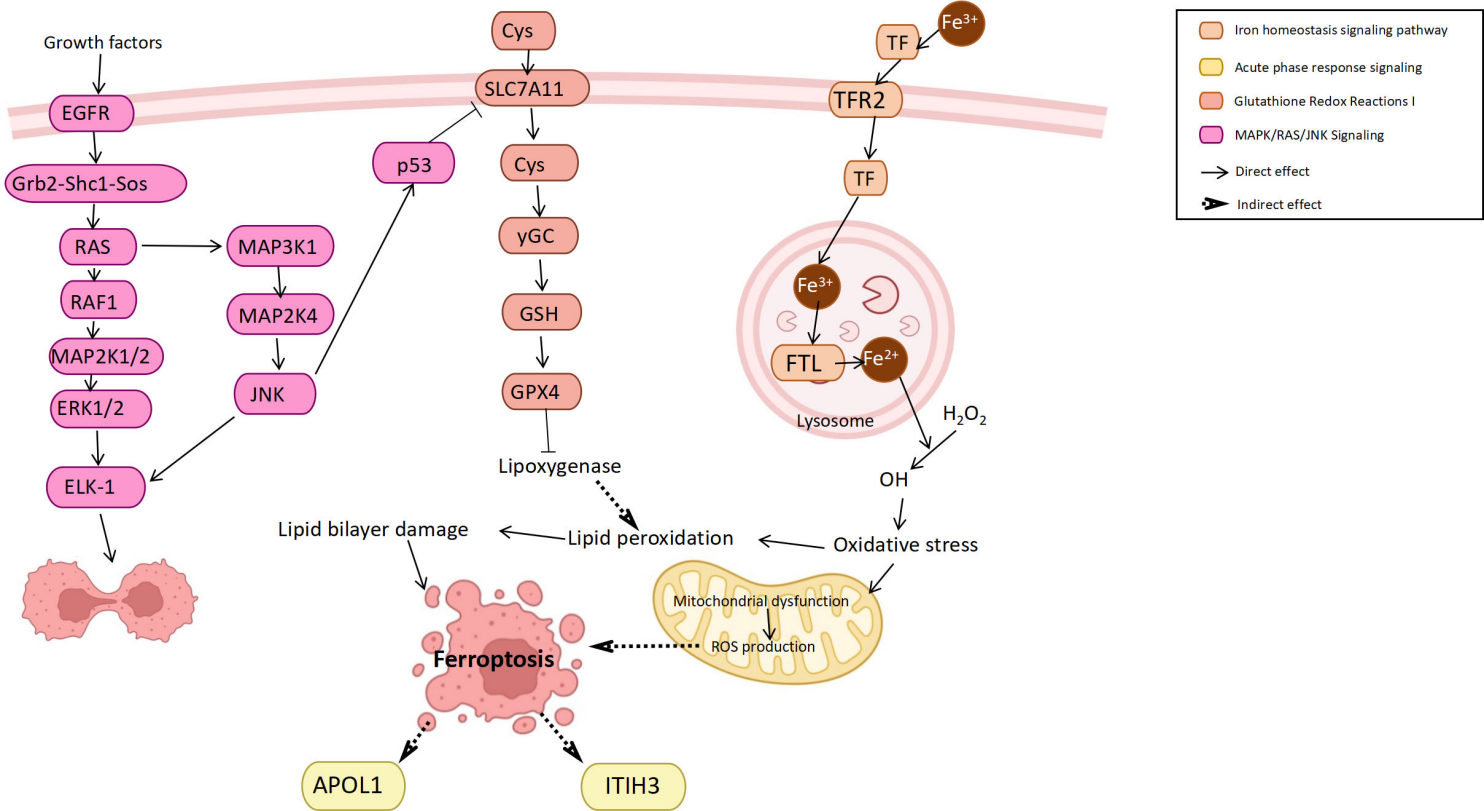
Mechanism of the pathophysiological process of bladder cancer.

In summary, we attempted to infer the connections between the outcomes in combination with pathway and functional analyses. The molecular interactions involved in these pathways complete the biological behaviors of bladder cancer cells, including proliferation, movement, metabolism, death and signal transduction (**Figure 5**).

### 4.2 Potential biomarkers for bladder cancer

After differential analysis of the healthy control group, cystitis group, and upper urinary tract cancer group, we took the common differential proteins among the groups and obtained a total of 8 proteins as candidate biomarkers. Among these candidate biomarkers, we further selected the downstream products of acute response signaling, APOL1 and ITIH3, to form a panel.

Apolipoprotein 1 (APOL1), the product of the human APOL1 gene, plays a role in lipid exchange and transport throughout the body and simultaneously regulates programmed cell death and autophagy (49). Changes in APOL1 function caused by various factors can lead to lipid disorders, cancer and other diseases (50). Studies have confirmed that APOL1 promotes the proliferation and invasion of pancreatic cancer cells by activating the NOTCH1 signaling pathway (51). In addition, APOL1 is highly expressed in head and neck squamous cell carcinoma tissues (52) and thyroid cancer tissues (53). At present, research on the relationship between APOL1 and bladder cancer mainly uses the TCGA database to conduct bioinformatics analysis for prognostic evaluation (54) and autophagy-related functions (55). In our study, the content of APOL1 in the bladder cancer, cystitis and upper urinary tract cancer samples was higher than that in the healthy control samples, suggesting that the protein is highly expressed in the urine of patients with urinary diseases. Combined with the relationship between APOL1 and other malignancies, we speculated that APOL1 may affect proliferation, invasion and autophagy in bladder cancer, thus affecting prognosis.

Inter-α-trypsin inhibitor heavy chain H3 (ITIH3) is a member of the inter-α-trypsin inhibitor family of proteins and is regarded as a carrier of hyaluronic acid in serum to localize, synthesize and degrade hyaluronic acid in cells (56). Currently, the inter-α-trypsin inhibitor family proteins play a particularly important role in inflammation and carcinogenesis (57). Studies have shown that ITIH3 is associated with the occurrence of colorectal cancer, gastric cancer, and endometrial cancer (58–60) and can be used as a biomarker for the screening of these malignant tumors. In our study, ITIH3 levels were higher in the bladder and upper urinary tract carcinoma groups than in the cystitis and healthy control groups, suggesting that ITIH3 may be highly expressed in the urine of urothelial carcinoma patients and that high levels of ITIH3 may regulate the occurrence of bladder cancer.

## 5 Conclusion

By analyzing the urine proteomes of bladder cancer and control group volunteers by LC-MS, we established a panel composed of two proteins as a potential biomarker for early bladder cancer. Through functional analysis of differential proteins, we deduced that the pathophysiological process of bladder cancer was mainly related to the ERK/MAPK signaling pathway, ferroptosis pathway, and iron homeostasis signaling pathway and regulated glutathione redox. Although the cross-talk between urine proteins and BC is still unclear, our findings may be helpful in further proteomic research. The results of our study indicated that proteins in urine could reflect changes in BC.

Many problems remain to be solved in future work. First, the samples in this study were from a single center, so different bladder cancer and healthy control samples from multiple centers should be collected for large-scale analysis to verify the conclusions. Moreover, the current standard of care of the patients should be focused in order to compare the performance with new biomarkers. Second, patients with a history of BC should be included in the study to monitor for BC recurrence. In addition, the impact factors of the urine proteome should be evaluated. Finally, molecular biology and related animal models should be used to help elucidate the possible mechanisms of proteins in early bladder cancer.

## Data availability statement

The datasets presented in this study can be found in online repositories. The names of the repository/repositories and accession number(s) can be found in the article/**Supplementary Material**.

## Ethics statement

The samples used in this study were from the Biobank of China-Japan Union Hospital of Jilin University. The consent procedure and research protocol for this study were approved by the Human and Animal Research Ethics Committee of China-Japan Union Hospital of Jilin University (No. 2023053015). The research methods met the standards set out in the Declaration of Helsinki.

## Author contributions

XZ: Writing – original draft, Writing – review & editing. FX: Data curation, Formal Analysis, Writing – original draft. TL: Investigation, Writing – original draft. JX: Validation, Writing – original draft. SY: Conceptualization, Writing – original draft. SZ: Writing – original draft. HL: Supervision, Writing – review & editing. CH: Funding acquisition, Supervision, Writing – review & editing.

## Supplementary material

Sheet T1 Differential Proteins in each cohort.

Sheet T2 Pathway and Function of each cohort.

Sheet T3 Result of ELISA.
