# Supplementary material for: Exploration of potential biomarkers for early bladder cancer based on urine proteomics
Source: Front Oncol. 2024 Feb 12;14:1309842. doi: 10.3389/fonc.2024.1309842 (PMC10894981; doi:10.3389/fonc.2024.1309842)

FIGURE S1: Quality control of data. A: Correlation of QC samples. B: Protein content in different groups. C: Abundance of blood contamination-related proteins in each sample. D: Abundance of cell debris contamination-related proteins in each sample.


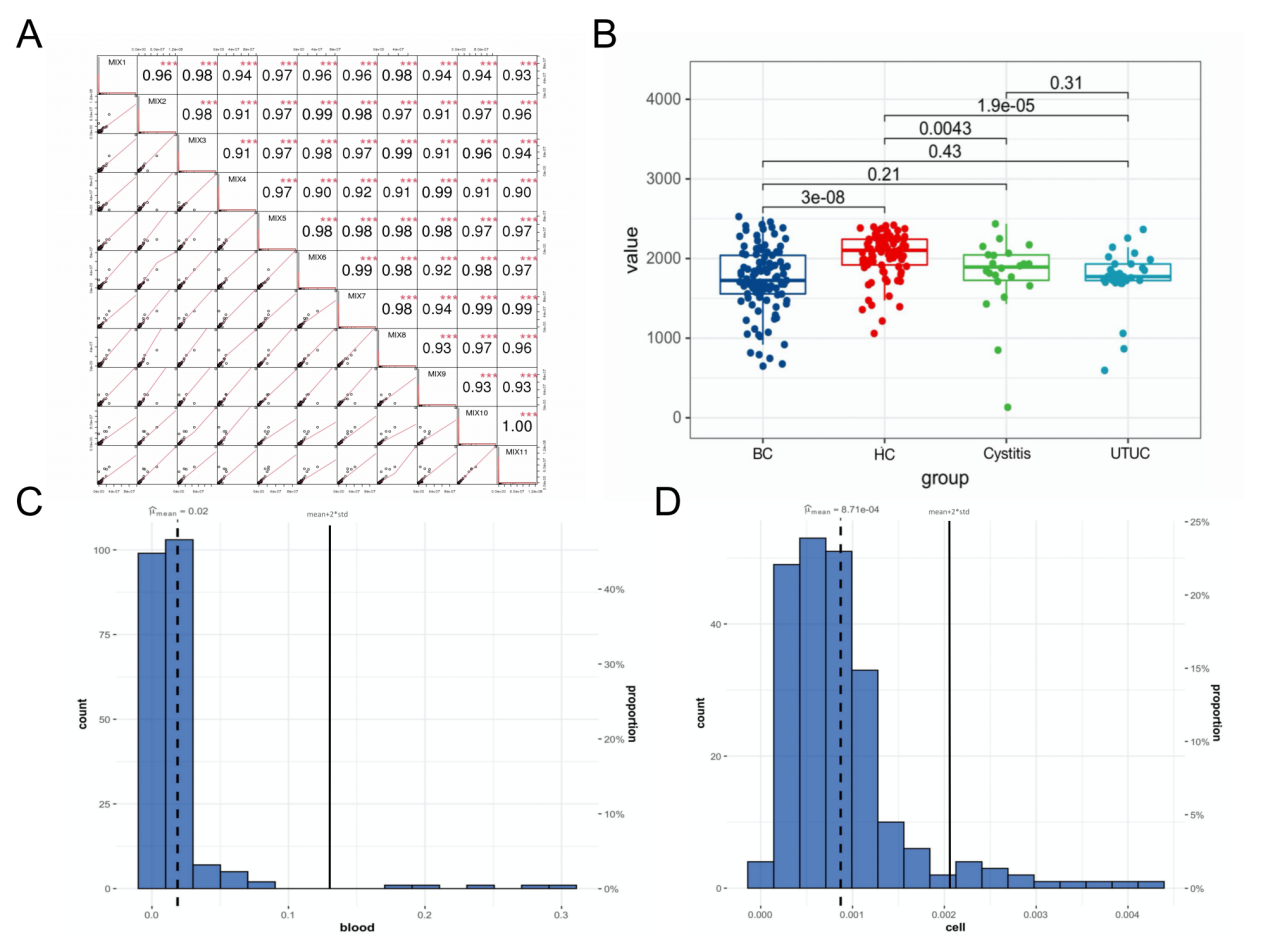


FIGURE S2: Results of OPLS-DA and permutation tests in each cohort(A,D: BC vs HC. B,E: BC vs Cystitis. C,F BC vs UTUC). A-C: Cluster analysis in each cohort. D-F: 100 permutation tests in each cohort.


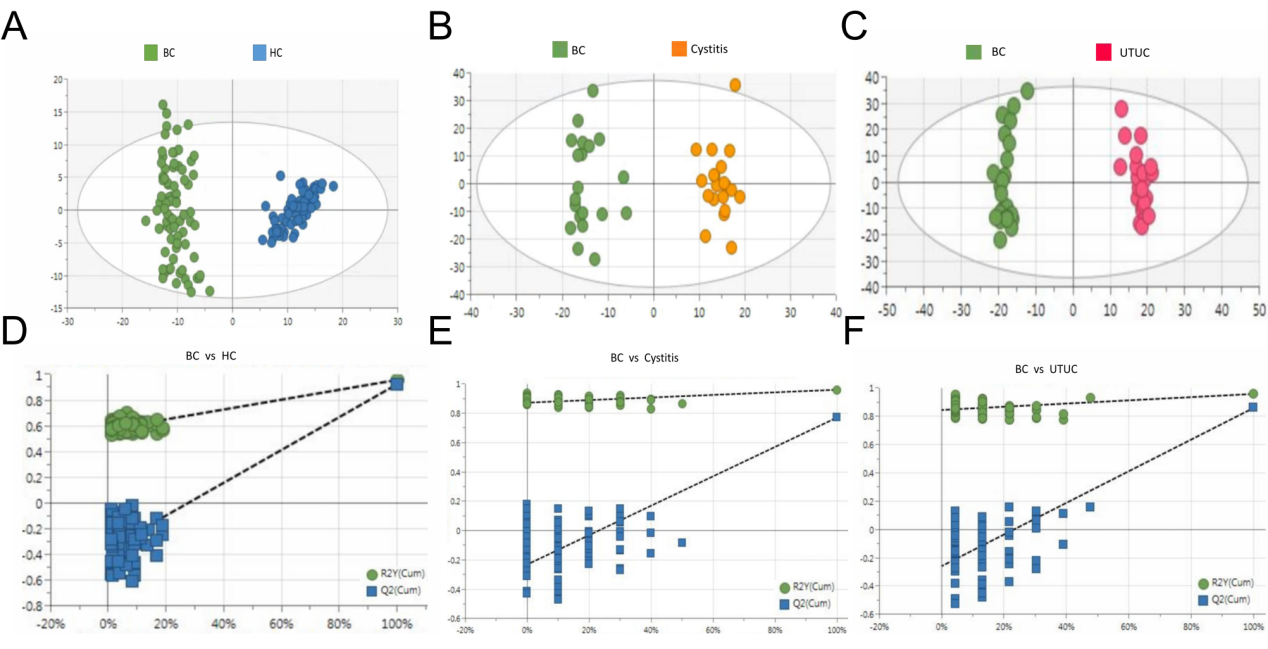


FIGURE S3: Biomarkers of BC and protein expression. A: Common differential proteins among different cohorts. B: Prediction effect of validation group. C-D: Protein content in each group(a: BC, b: HC, c:Cystitis, d: UTUC).


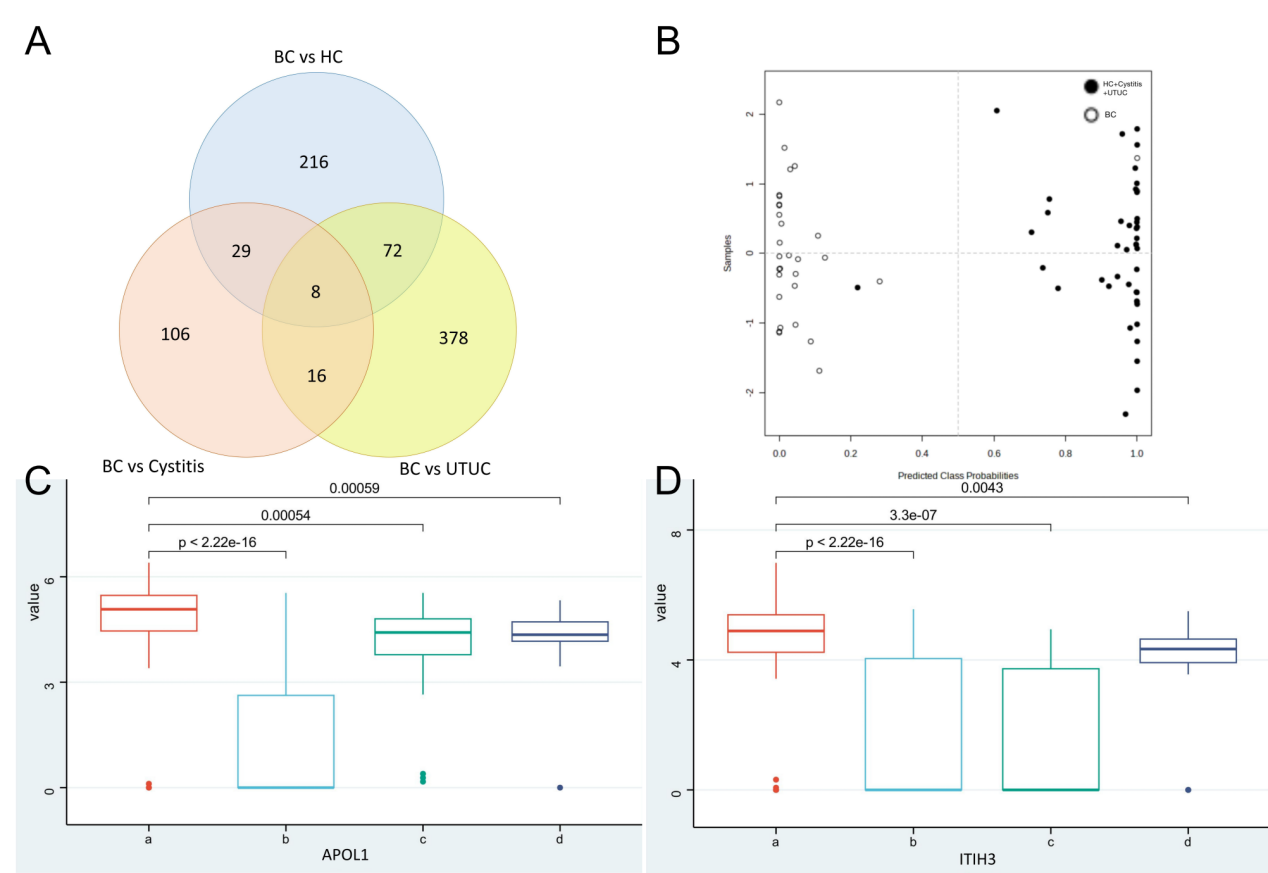

Supplement: Sheet T1 — Differential Proteins in each cohort. [file DataSheet_1.zip › Supplementary Files/Supplementary Figures.docx]
