# Supplementary figures and images for: Exploration of potential biomarkers for early bladder cancer based on urine proteomics
Source: Front Oncol. 2024 Feb 12;14:1309842. doi: 10.3389/fonc.2024.1309842 (PMC10894981; doi:10.3389/fonc.2024.1309842)

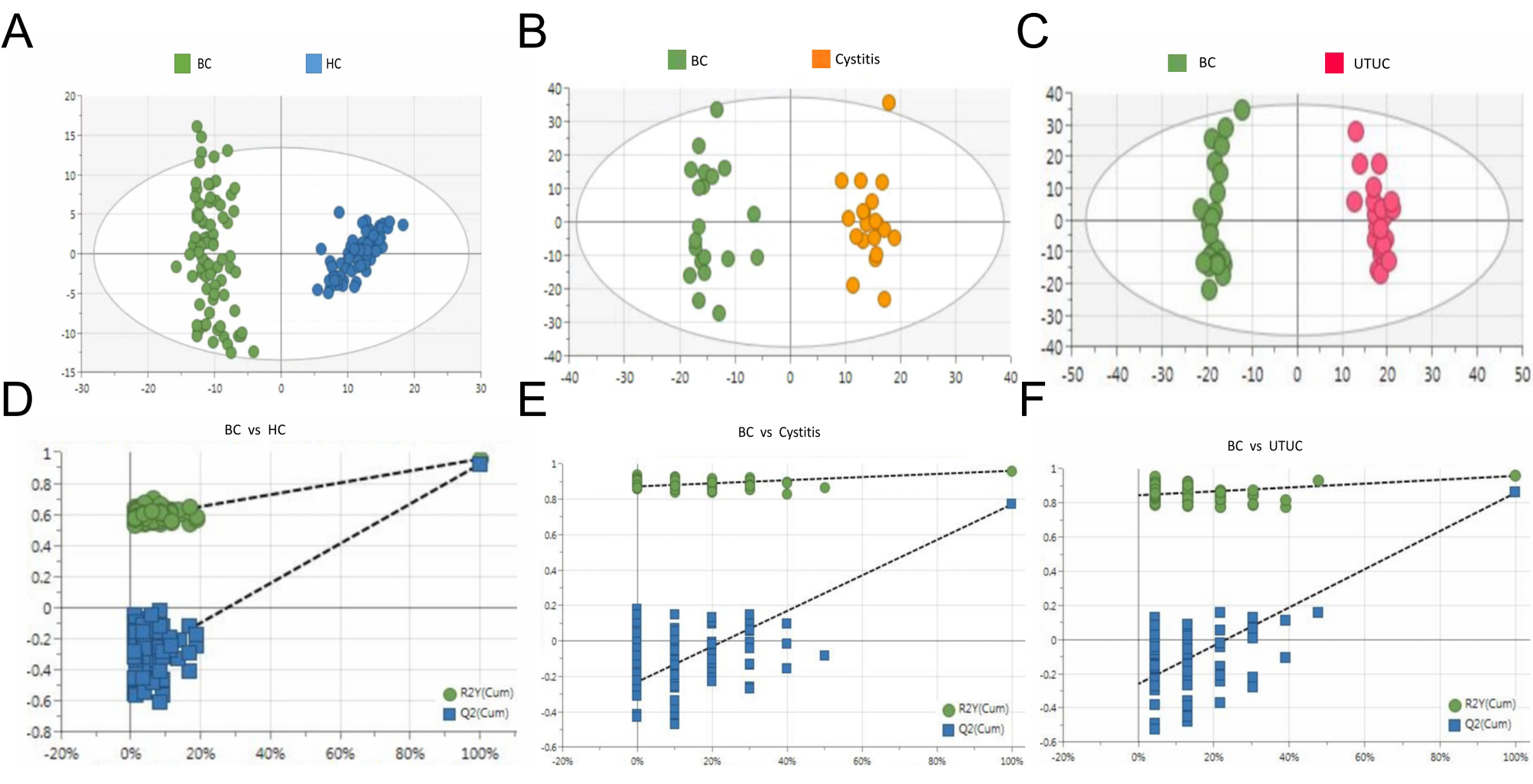

Supplement: Sheet T1 — Differential Proteins in each cohort. [file DataSheet_1.zip › Supplementary Files/FIGURE S2.tif]

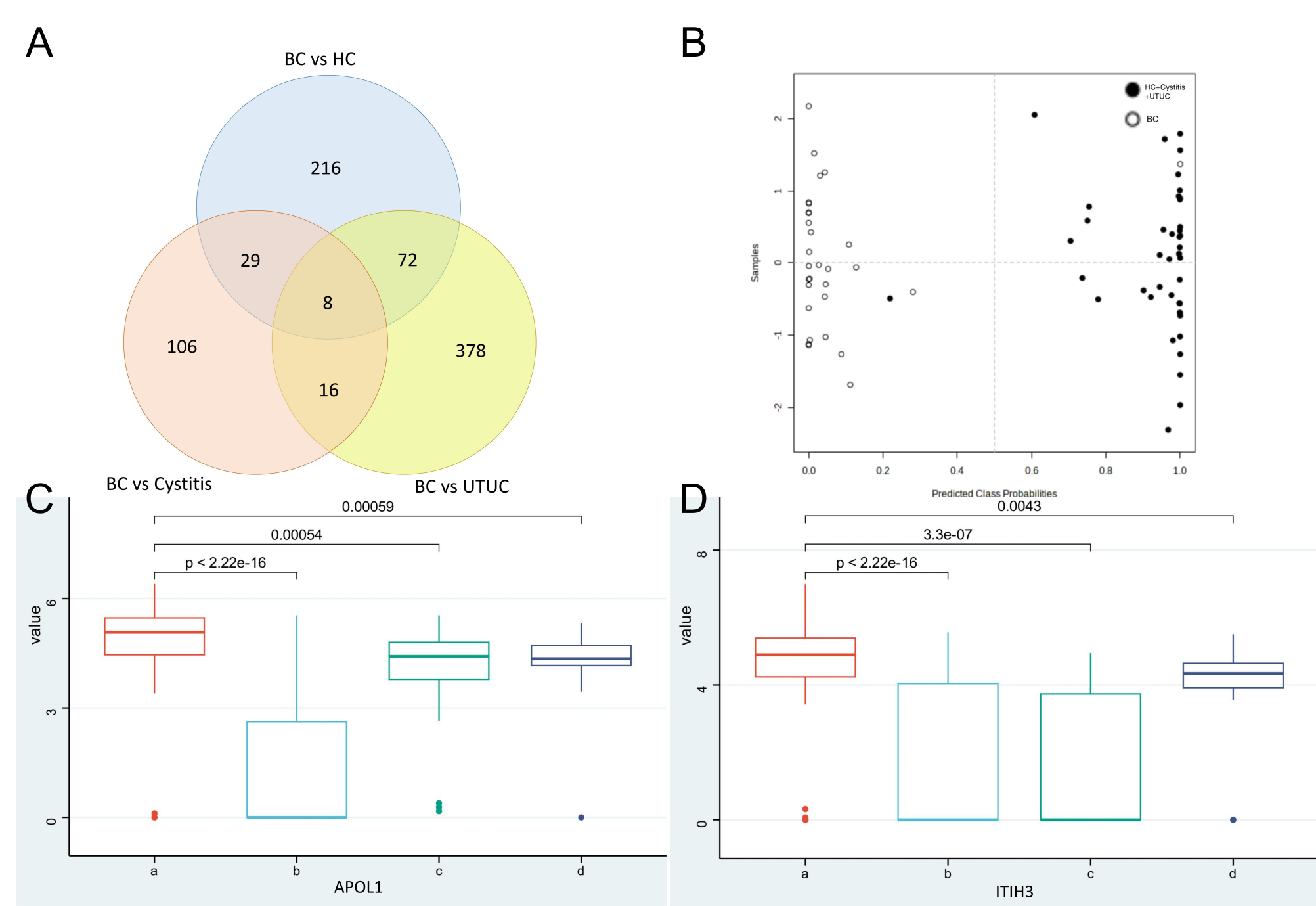

Supplement: Sheet T1 — Differential Proteins in each cohort. [file DataSheet_1.zip › Supplementary Files/FIGURE S3.tif]

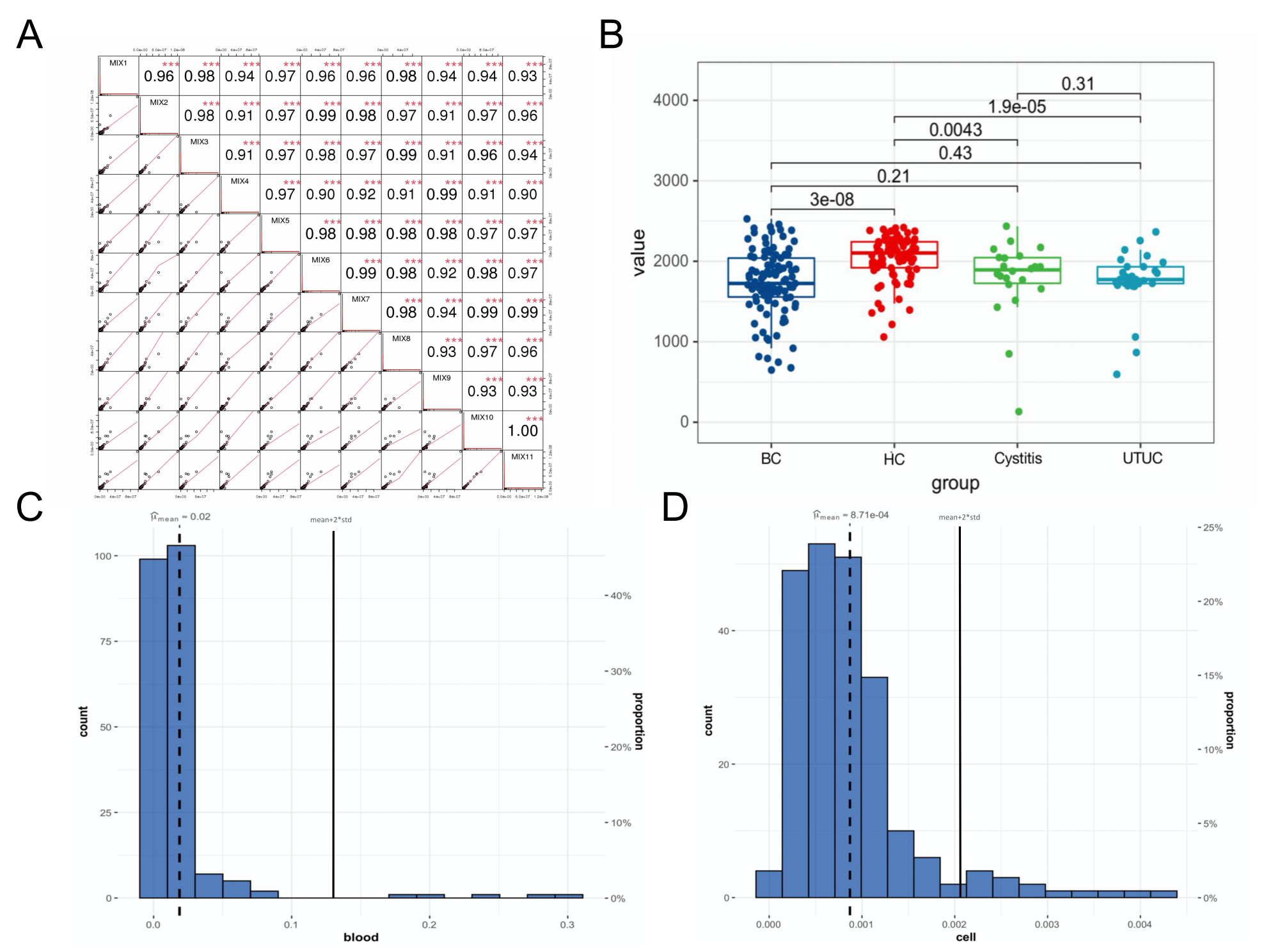

Supplement: Sheet T1 — Differential Proteins in each cohort. [file DataSheet_1.zip › Supplementary Files/FIGURE S1.tif]
